# Ultrasound-based radiomics and habitat analysis for noninvasive assessment of Ki-67 overexpression in breast cancer

**DOI:** 10.3389/fonc.2026.1803826

**Published:** 2026-04-29

**Authors:** Li Zhu, Shanni Dong, Xushuang Qin, Xiaoying Mi, Jiaqi Zhang, Xiaoshu Zhu, Yuting Liu, Jiake Hua, Shuangxi Chen

**Affiliations:** 1 Department of Ultrasound Medicine, Zhejiang Provincial People’s Hospital, Hangzhou, China; 2 Department of Interventional Medicine, Zhejiang Provincial People’s Hospital, Hangzhou, China; 3 The Second Clinical Medical College, Hangzhou Normal University Affiliated Hospital, Hangzhou, China

**Keywords:** breast cancer, habitat, Ki-67, radiomics, ultrasound

## Abstract

**Background:**

Accurate preoperative assessment of Ki-67 proliferation index remains a clinical challenge in breast cancer management. Conventional ultrasound radiomics often fails to fully capture intratumoral heterogeneity, suffers from overfitting, and includes redundant features that limit generalizability.

**Methods:**

In this retrospective study, we analyzed preoperative ultrasound images and immunohistochemical results from 288 women with pathologically confirmed breast cancer. We extracted both conventional radiomic features and intratumoral habitat features, computed risk scores, and integrated them with clinicopathological variables (e.g., progesterone receptor status, lymph node involvement) to construct a nomogram. Model performance was assessed by area under the receiver operating characteristic curve (AUC), calibration curves, and decision curve analysis (DCA).

**Results:**

The Clinics_Habitat_Radiomics model achieved AUCs of 0.877 (95% CI: 0.826–0.929) in the training cohort and in the validation cohort, the model achieved an AUC of 0.830, with a sensitivity of 60.3% and specificity of 91.7%, significantly outperforming other models. Calibration curves indicated close agreement between predicted probabilities and observed outcomes (Hosmer–Lemeshow test: *p* = 0.14 [training], *p* = 0.19 [validation]). DCA demonstrated superior net clinical benefit across a range of threshold probabilities compared with single-modality approaches.

**Conclusions:**

The integration of habitat analysis with ultrasound-based radiomics enables the development of a nomogram that synergistically incorporates multimodal imaging features and clinicopathological parameters, offering a non-invasive predictive tool for Ki-67 expression in breast cancer. This model not only enhances the precision of tumor biology assessment but also provides actionable insights for optimizing therapeutic regimens, monitoring treatment responses, and stratifying prognostic risks, thereby bridging the gap between radiomic diagnostics and personalized oncology care.

## Introduction

Breast cancer is the most frequently diagnosed malignancy and the fifth leading cause of cancer-related death among women worldwide (1). The Ki-67 nuclear protein, a well-established marker of cell proliferation, plays a key role in regulating cellular growth and differentiation, and serves as an independent prognostic factor in multiple cancers, including breast cancer, lung cancer, and lymphoma (2). Currently, clinical assessment of Ki-67 relies on immunohistochemistry of biopsy specimens. This invasive approach is constrained by single-site sampling, which fails to capture intratumoral spatial heterogeneity and precludes dynamic monitoring of proliferation activity.

In recent years, Radiomics is widely applied in differential diagnosis and prognosis prediction (3, 4). Ultrasound radiomics has emerged as a pivotal tool in breast cancer research, offering a non-invasive avenue to predict molecular markers such as Ki-67, which are critical for prognostic stratification but traditionally require invasive biopsies prone to sampling bias. While conventional radiomic models extract high-throughput features to enhance quantitative analysis (5), they predominantly characterize tumors at a macroscopic level, often failing to capture the localized phenotypic variations that drive intratumoral heterogeneity (ITH) (6). Recent studies highlight the limitations of standard computer-aided detection in resolving these micro-environmental complexities (7, 8). Habitat imaging addresses this gap by clustering pixels and voxels with similar imaging phenotypes to delineate biologically distinct subregions, thereby quantifying textural and spatial topological relationships within the lesion (9, 10). Unlike MRI-based habitat analysis, which is susceptible to motion artifacts and high costs, ultrasound-based habitat imaging leverages high spatial resolution and real-time capabilities to provide a more robust and accessible characterization of tumor heterogeneity. This approach enables a precise mapping of the tumor microenvironment, potentially serving as an imaging biomarker for Ki-67 expression and guiding personalized therapeutic strategies.

In this study, we propose an innovative integration of habitat analysis with ultrasound radiomics to non-invasively predict preoperative Ki-67 expression status in breast cancer. Our aim is to establish an imaging-based biomarker that can facilitate optimized patient stratification for targeted and immunotherapy selection.

## Materials and methods

### Patients

This retrospective study was approved by the Ethics Committee of our institution (Approval No. QT2022328). Owing to its retrospective design, the requirement for written informed consent was waived. Between January 2021 and January 2025, a total of 353 women with breast cancer confirmed by surgical pathology were initially identified.

Inclusion criteria were as follows: (1) preoperative ultrasound examination with adequate image quality; (2) histopathologically confirmed malignant breast tumor; (3) availability of complete immunohistochemical results, including Ki-67 expression status; and (4) comprehensive clinical data. Exclusion criteria comprised: (1) prior radiotherapy, chemotherapy, or other neoadjuvant treatment before ultrasound examination; (2) history of breast implant placement; and (3) coexisting malignancies at other sites.

**Figure 1:**
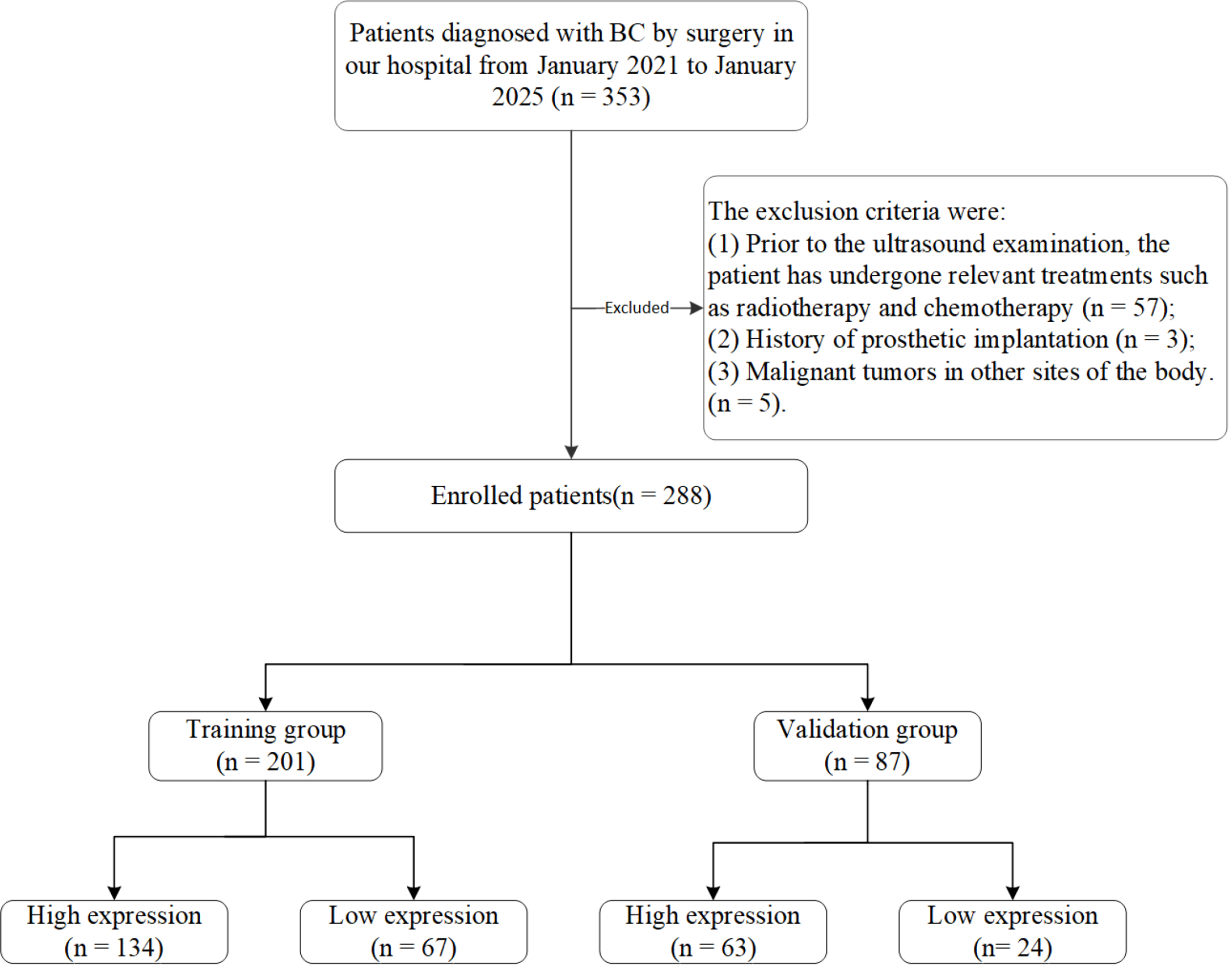
A flowchart of participant enrollment.

Following application of these criteria, 288 patients were eligible for inclusion. They were randomly allocated to a training cohort (n = 201, 70%) and a validation cohort (n = 87, 30%) in a 7:3 ratio for model development and internal validation. The patient screening and enrollment process is illustrated in **Figure 1**.

### Ultrasound image acquisition

Breast ultrasound examinations were performed using three commercially available color Doppler systems: the GE LOGIQ E9 (GE Healthcare, USA), Siemens Acuson Sequoia (Siemens Healthineers, Germany), and Mindray DC-8 (Mindray Medical International, China). Images were acquired with a high-frequency linear array transducer (center frequency: 7–10 MHz), capturing the largest cross-sectional view of the breast mass and corresponding color Doppler flow imaging (CDFI). All images were stored in the Digital Imaging and Communications in Medicine (DICOM) standard format to ensure data integrity and traceability for subsequent analysis.

### Ultrasound image analysis

Image acquisition followed the 2013 Breast Imaging Reporting and Data System (BI-RADS) guidelines of the American College of Radiology (ACR) (11). Collect the following ultrasonographic features of tumors: Maximum tumor diameter (mm), Shape, Margin, Echo Pattern, Posterior Features, Calcifications, Orientation, Echogenic rind, and Adler classification. Assess tumor vascularity using the Adler blood flow grading system (12):

Grade 0: No detectable intratumoral blood flow signals.

Grade I: 1–2 punctate or short linear vascular signals within the lesion.

Grade II: 3–4 punctate vascular signals or one longer vascular structure penetrating the tumor.

Grade III: ≥5 punctate vascular signals, two or more elongated vascular structures, or interconnected vascular networks forming a reticular pattern.

Two attending physicians with ≥9 years of experience in breast ultrasound independently evaluated the ultrasound features. In cases of disagreement, a consensus was reached through discussion.

### Baseline evaluation

Baseline demographic characteristics, tumor parameters, and clinicopathological data were collected, including age, sex, estrogen receptor (ER), progesterone receptor (PR), human epidermal growth factor receptor 2 (HER-2), lymph node metastasis (LMN), and menopausal status.

### Pathological examination

Ki-67 expression levels were determined by immunohistochemical staining of surgical specimens, performed and analyzed by a senior attending pathologist. The diagnostic criterion for positive Ki-67 expression was the percentage of tumor cell nuclei stained in hotspot fields of the section. According to the 2013 St. Gallen consensus, in which the majority of experts supported a 20% threshold (13), patients were stratified into high Ki-67 expression (Ki-67 ≥ 20%) and low expression (Ki-67 < 20%) groups.

### Tumor segmentation and radiomic feature extraction

Preoperative ultrasound images in DICOM format were exported to the open-source software ITK-SNAP (version 3.8.0; http://www.itksnap.org/). Two breast sonographers, with 9 and 16 years of experience respectively, independently delineated whole-tumor regions of interest (ROIs) on each image. If multiple lesions were present, only the largest was selected for analysis. Inter-observer reproducibility was assessed using the intraclass correlation coefficient (ICC) (14); an ICC > 0.8 was considered indicative of good consistency. Radiomic features were then extracted using the PyRadiomics platform (version 4.0), encompassing first-order statistics (e.g., gray-level histogram), second-order texture features (e.g., gray-level co-occurrence matrix, gray-level run-length matrix), and higher-order features (e.g., morphological parameters after wavelet transformation). A total of 1,312 original radiomic features were obtained from the tumor region. Apply minimum-maximum normalization to all features and use robust radiomics features that are insensitive to changes in absolute intensity.

### Habitat imaging analysis

Local entropy and energy values were extracted from individual voxels within each ROI, generating feature vectors that captured distinct dimensions of voxel properties. The optimal number of clusters was determined as four using the Calinski-Harabasz (CH) index, and K-means clustering was applied to partition the tumor into intratumoral subregions (15). Nine hundred twenty-nine features were computed for each habitat, yielding a total of 3,716 habitat features (929 × 4 clusters) across the four habitats.

### Feature selection and model construction

This retrospective analysis included data from 288 patients. All radiomic features underwent comprehensive preprocessing. Continuous variables were scaled to the [0,1] interval using min–max normalization, and outliers were addressed by median imputation (16). Clinical variables were dimensionally reduced via logistic regression to optimize computational efficiency and enhance interpretability.

**Figure 2:**
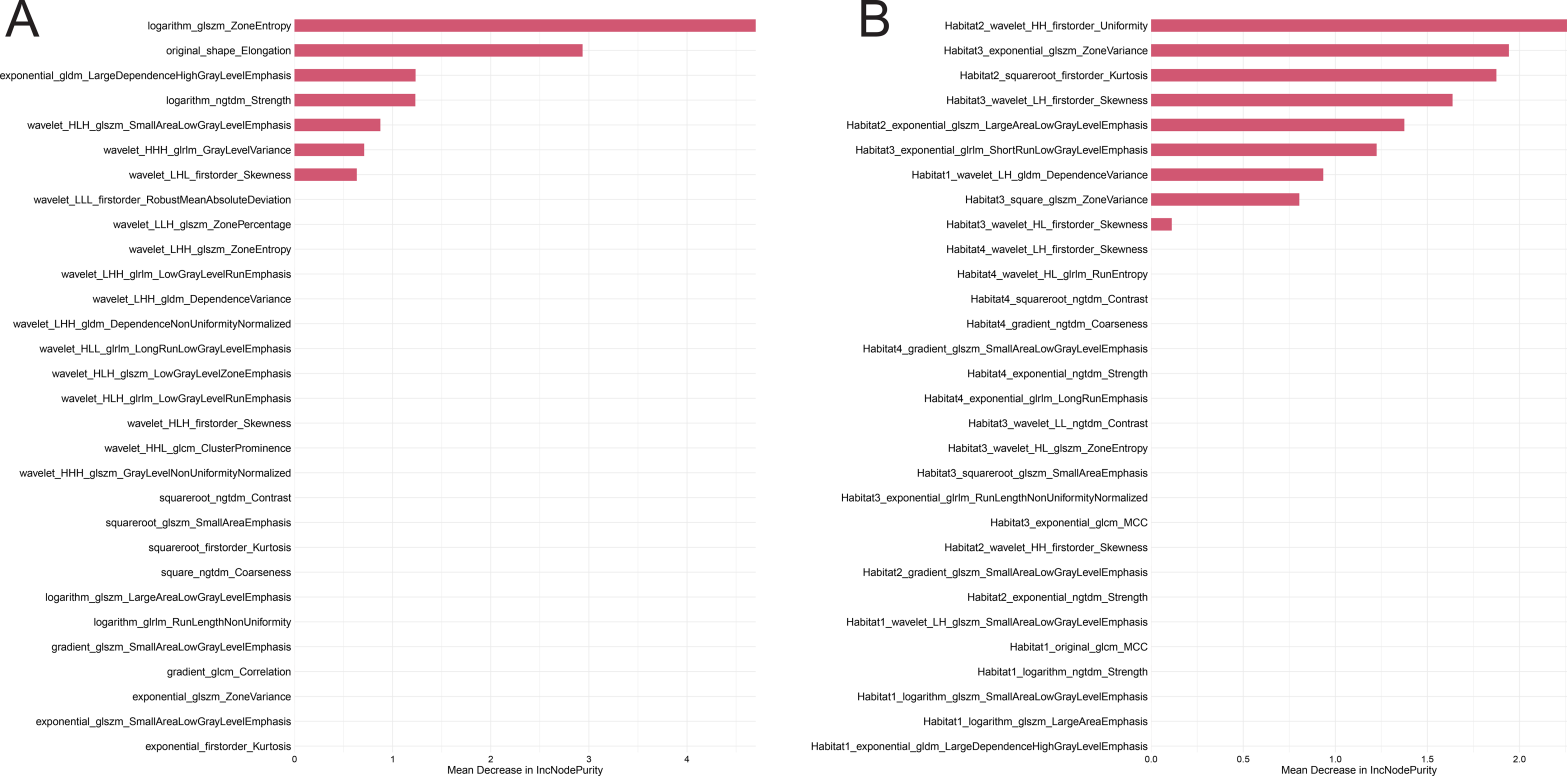
SHAP values of radiomic features selected by random forest. **(A)** Habitat features. **(B)** Radiomic features.

From the initial 1,312 tumor-specific radiomic features, the minimum redundancy maximum relevance (mRMR) algorithm was applied to select the 30 most informative habitat features for each modality to mitigate overfitting. These 30 selected features were subsequently used in their entirety to construct the random forest model. Notably, the random forest classifier was configured with three decision trees (number of trees = 3) for the final model construction (**Figure 2**).The entire cohort was divided into a training set (n = 201) and a validation set (n = 87) at a 7:3 ratio. Selected radiomic features were visualized and interpreted using Shapley additive explanation (SHAP) to illustrate each feature’s contribution to the predicted probability. The resulting output, designated as the Rad-score, represented the risk value of the tumor radiomic model.

**Figure 3:**
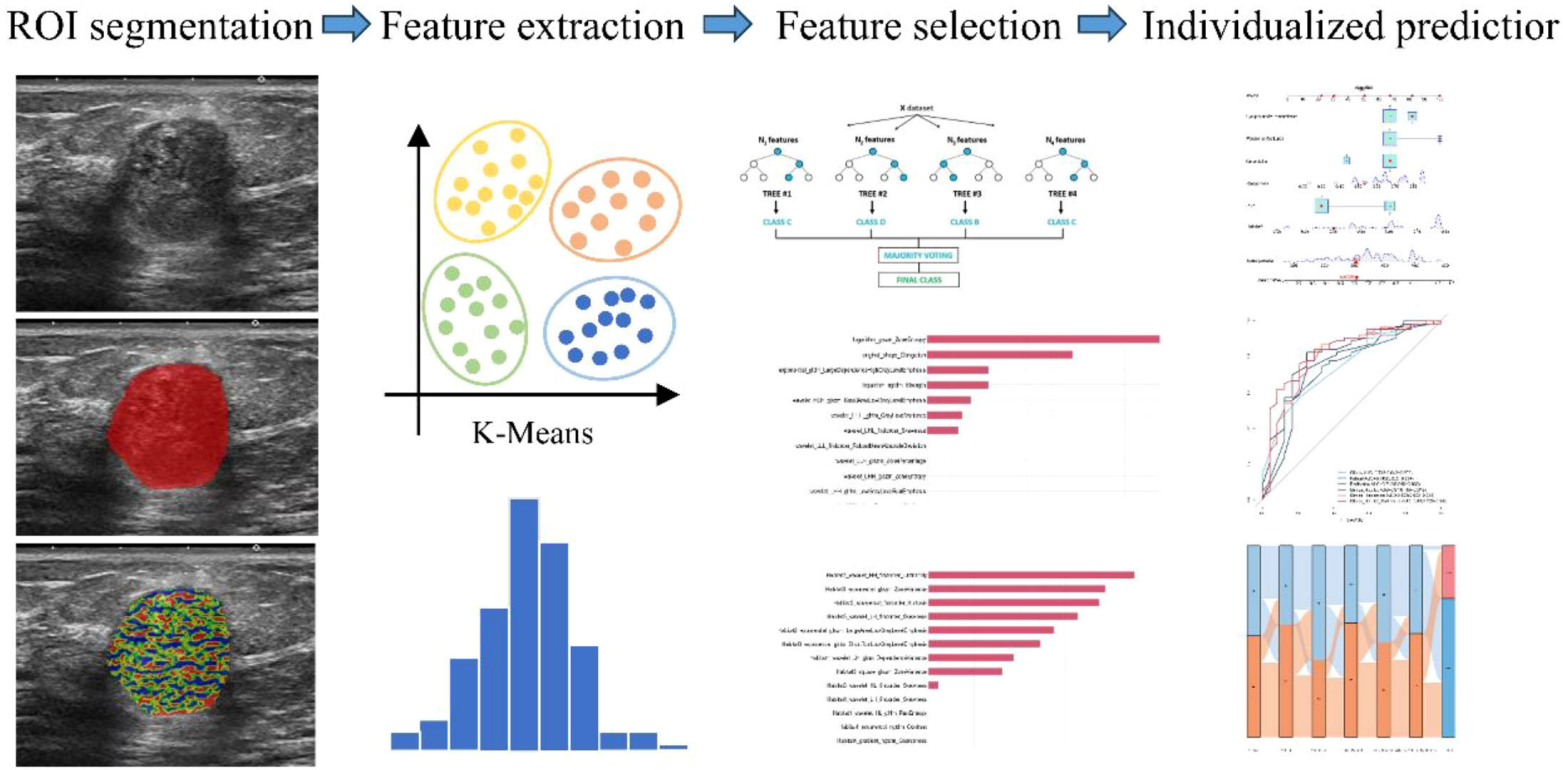
Schematic overview of the habitat imaging methodology.

The Rad-score was then integrated with habitat features and clinically significant variables identified by logistic regression to construct a nomogram for visual model representation. Inter-variable correlations within the combined model were analyzed. This hybrid architecture fused imaging features with clinical parameters. The complete workflow of model development is illustrated in the **Figure 3**.

### Statistical analysis

All statistical analyses were performed using R software (version 4.4.2; http://www.r-project.org). Continuous variables were compared using Student’s t-test or the Mann–Whitney U test, and categorical variables were analyzed with the chi-square test or Fisher’s exact test, as appropriate. Model performance was assessed by receiver operating characteristic (ROC) curve analysis, and the area under the curve (AUC), Decision curve analysis (DCA) was conducted to evaluate clinical utility across a range of threshold probabilities. A two-sided P value of <0.05 was considered statistically significant.

## Result

### Patient characteristics

A total of 288 patients were included and divided into a training cohort (n = 201) and a validation cohort (n = 87). Detailed baseline characteristics of the two cohorts are summarized in **Table 1**. No significant differences were observed between the training and validation cohorts in terms of clinical or ultrasound characteristics (all p > 0.05).

**Table 1:** Clinical information of patients in training and validation sets.

Characteristics	Overall	Training group	Validation group	P-value
n	288	201	87	
Age, mean (SD)	55.2(10.8)	55.8(10.6)	53.9(11.2)	0.196
ER, n (%)				0.931
Negative	90(31.2)	62(30.8)	28(32.2)	
Positive	198(68.8)	139(69.2)	59(67.8)	
PR, n (%)				0.489
Negative	113(39.2)	82(40.8)	31(35.6)	
Positive	175(60.8)	119(59.2)	56(64.4)	
Her-2, n (%)				0.486
Negative	62(21.5)	46(22.9)	16(18.4)	
Positive	226(78.5)	155(77.1)	71(81.6)	
Lymph Node Metastasis, n (%)				0.662
No	195(67.7)	134(66.7)	61(70.1)	
Yes	93(32.3)	67(33.3)	26(29.9)	
Diameter(mm), mean (SD)	23.1(14.2)	22.9(12.4)	23.7(17.7)	0.712
Shape, n (%)				0.640
Oval or round	8(2.8)	3(1.5)	2(2.3)	
Irregular	280(97.2)	198(98.5)	85(97.7)	
Margin, n (%)				0.458
Circumscribed	29(10.1)	18(9.0)	11(12.6)	
Not circumscribed	259(89.9)	183(91.0)	76(87.4)	
Echo Pattern, n (%)				0.699
Hypoechoic	38(13.2)	25(12.4)	13(14.9)	
Heterogeneous	250(86.8)	176(87.6)	74(85.1)	
Posterior Features, n (%)				0.058
None/Enhancement	238(82.6)	160(79.6)	78(89.7)	
Shadowing	50(17.4)	41(20.4)	9(10.3)	
Calcifications, n (%)				0.666
No	155(53.8)	106(52.7)	49(56.3)	
Yes	133(46.2)	95(47.3)	38(43.7)	
Orientation, n (%)				0.053
Parallel	253(87.8)	182(90.5)	71(81.6)	
Not parallel	35(12.2)	19(9.5)	16(18.4)	
Echogenic rind, n (%)				0.999
No	197(68.4)	137(68.2)	60(69.0)	
Yes	91(31.6)	64(31.8)	27(31.0)	
Adler classification, n (%)				0.608
Grade 0、I	203(70.5)	144(71.6)	59(67.8)	
Grade II、III	85(29.5)	57(28.4)	28(32.2)	
Menopause, n (%)				0.375
No	188(65.3)	135(67.2)	53(60.9)	
Yes	100(34.7)	66(32.8)	34(39.1)	
Family history of cancer, n (%)				0.941
Negative	203(70.5)	142(70.6)	61(70.1)	
Breast cancer	25(8.7)	18(9.0)	7(8.0)	
Others positive	60(20.8)	41(20.4)	19(21.8)	

### Clinical feature dimensionality reduction and selection

A total of 16 clinical variables were collected and subjected to a rigorous feature selection process based on logistic regression. This analysis identified four clinically significant features exhibiting important associations (**Figure 2**): Progesterone Receptor (PR) status, Lymph Node Metastasis (LMN), Orientation, and Posterior Features. A clinical risk score was then calculated using logistic regression analysis, and the clinical feature model was established accordingly.

### Model construction for prediction of Ki-67

The inter-observer ICC for radiomic features was 0.917 ± 0.061; Observer 1’s intra-observer ICC, measured one month later, was 0.909 ± 0.028. Six predictive models were developed: a clinical feature model (Clinics), a habitat feature model (Habitat), an ultrasound radiomics model (Radiomics), a combined habitat–clinical model (Clinics_Habitat), a combined radiomics–clinical model (Clinics_Radiomics), and an integrated habitat–radiomics–clinical model (Clinics_Habitat_Radiomics).

### Model evaluation and visualization

**Figure 4:**
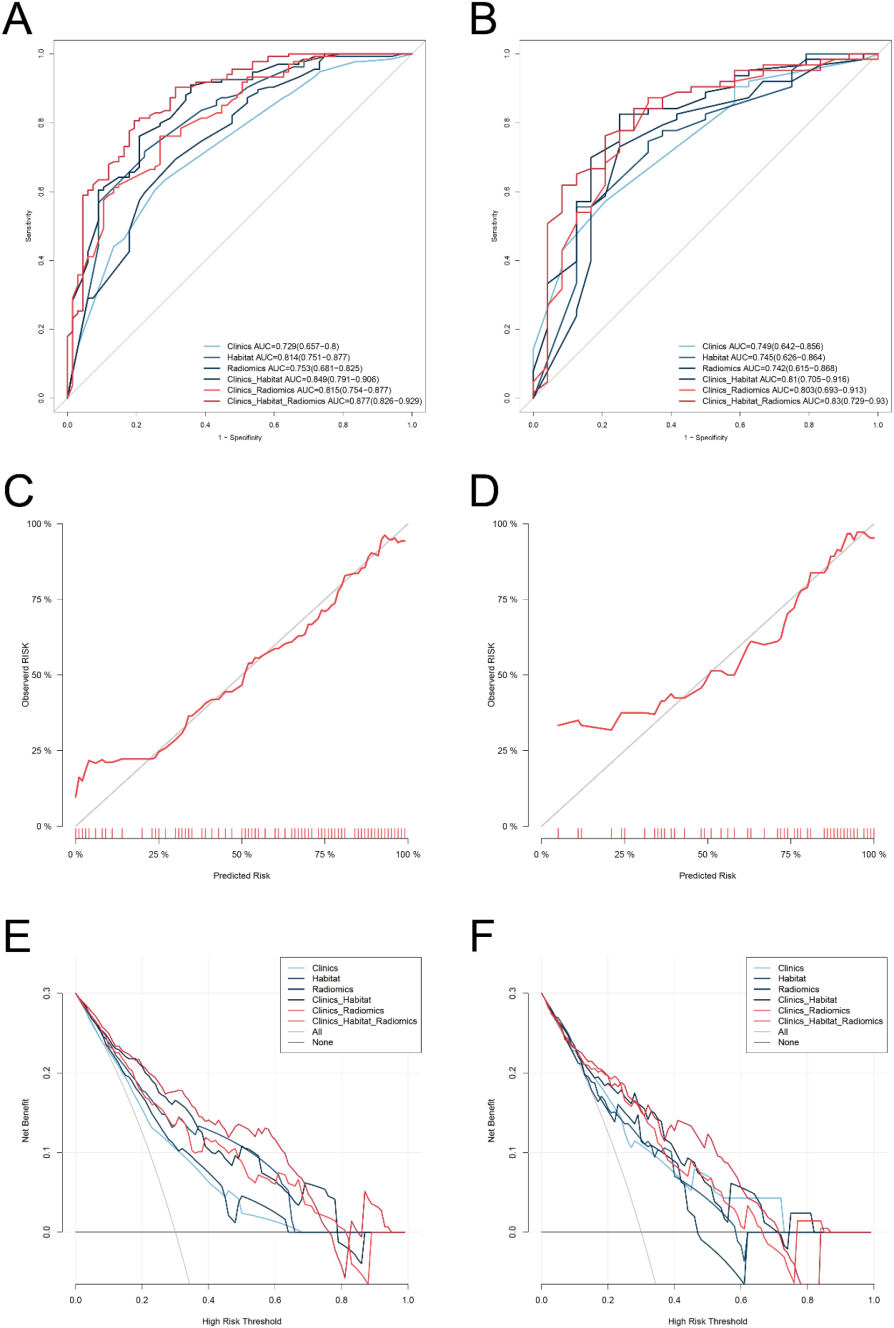
Receiver operating characteristic (ROC) curves, calibration curves, and decision curve analysis (DCA) for all models. Participants were divided into training **(A, C, E)** and validation **(B, D, F)** cohorts. **(A, B)** ROC curves in the training and validation cohorts. **(C, D)** Calibration curves in the training and validation cohorts. **(E, F)** DCA curves in the training and validation cohorts.

**Figure 5:**
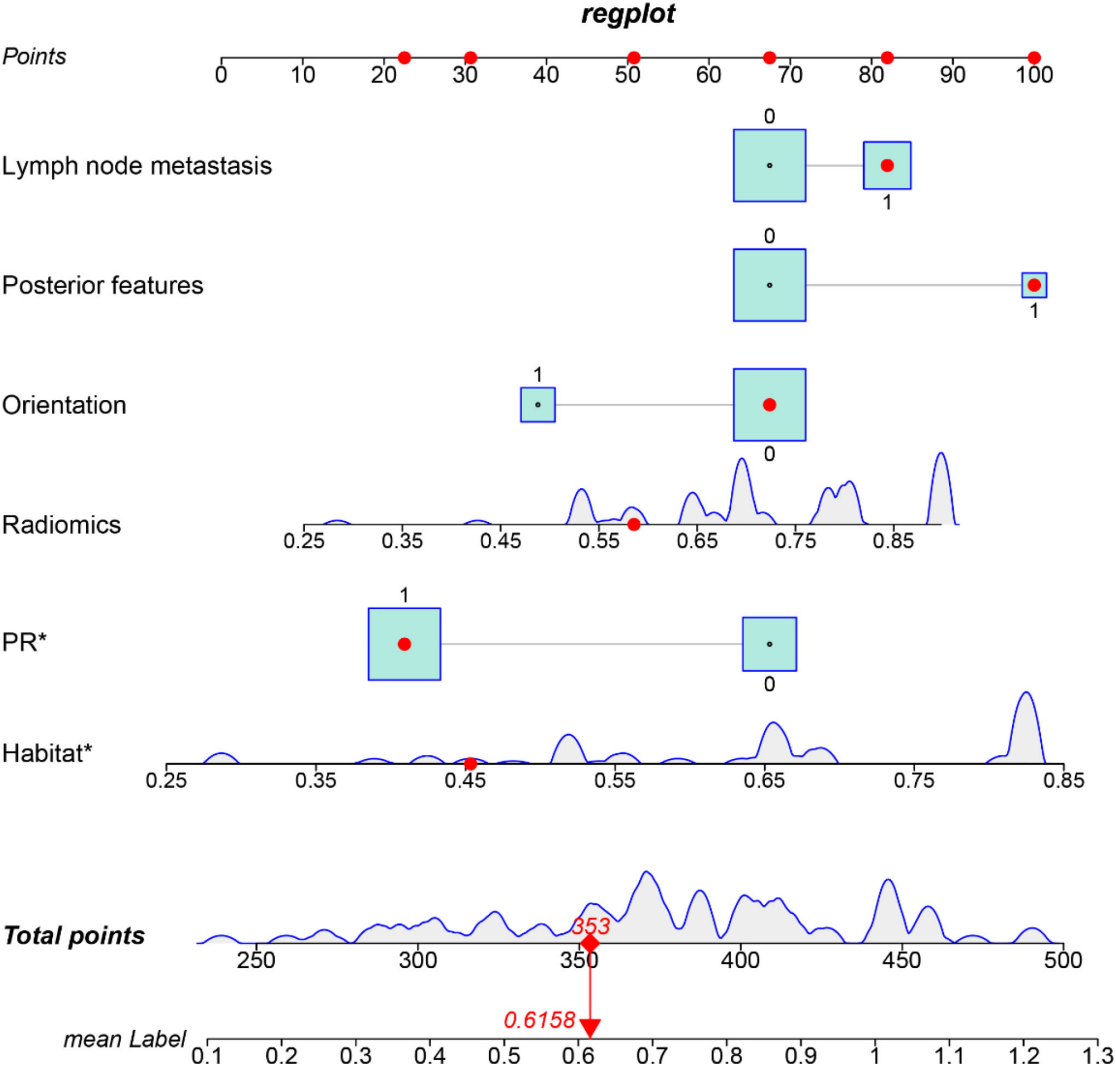
Nomogram for visualizing the integrated model and illustrating correlations among variables. A combined nomogram incorporating PR status, Lymph Node Metastasis (LMN), Orientation, Posterior Features, and Radiomics score was developed to predict the risk of Ki-67 positivity in patients with breast cancer. * Means p < 0.05.

Predictive performance of the clinical, habitat, and combined models was assessed by receiver operating characteristic (ROC) curve analysis. The Habitat model achieved areas under the ROC curve (AUC) of 0.814 (95% CI: 0.751–0.877) in the training cohort and 0.745 (95% CI: 0.626–0.864) in the validation cohort. The Clinics_Habitat_Radiomics model exhibited the best performance, with AUC of 0.877 (95% CI: 0.826–0.929) in the training cohort and 0.830 (95% CI: 0.729–0.930) in the validation cohort (**Figures 4A, B**). Based on these results, a clinically applicable nomogram was constructed (**Figure 5**).

Calibration curves demonstrated close agreement between predicted probabilities and observed outcomes (Hosmer–Lemeshow test: *p* = 0.14 for training cohort; *p* = 0.19 for validation cohort; **Figures 4C, D**). Decision curve analysis indicated that the combined model provided greater clinical net benefit across threshold probabilities of 20%–65% (training) and 40%–60% (validation) compared with single-modality models (**Figures 4E, F**).

Discriminatory performance of the Clinics_Habitat_Radiomics model was significantly higher in the training cohort than in the Clinics, Radiomics, Clinics_Habitat, and Clinics_Radiomics models (DeLong test: all |z| > 1.96, *p* < 0.05; **Supplementary Figure S1**), whereas no significant improvement was observed in the validation cohort.

## Discussion

Accurate preoperative prediction of Ki−67 expression status in breast cancer provides clinicians with valuable prognostic information and facilitates individualized treatment planning (17). In this study, we innovatively integrated ultrasound radiomics with habitat imaging analysis to develop a comprehensive model for predicting Ki−67 expression levels in breast cancer. Our results demonstrated that the ultrasound radiomic nomogram based on habitat analysis yielded excellent predictive performance in both the training cohort (area under the receiver operating characteristic curve [AUC], 0.877) and the validation cohort (AUC, 0.830), significantly outperforming models based solely on clinical variables or conventional radiomics features. These findings suggest a promising noninvasive approach for preoperative assessment of Ki−67 expression in breast cancer, potentially enhancing risk stratification and guiding therapeutic decision−making.

In the clinical feature model screening of this study, progesterone receptor (PR) status, lymph node metastasis (LMN), tumor orientation, and posterior features emerged as independent factors influencing Ki-67 expression levels in breast cancer. Notably, axillary lymph node metastasis and non-parallel growth of the tumor were identified as independent risk factors for high Ki-67 expression. High Ki-67 expression signifies active tumor cell proliferation, elevated malignant potential, and enhanced invasiveness—characteristics that drive tumor cells to breach normal tissue planes and infiltrate surrounding structures, leading to an “upright” growth pattern (18). This aggressive behavior also facilitates the spread of tumor cells to adjacent tissues, blood vessels, and lymphatic vessels, thereby increasing the likelihood of axillary lymph node metastasis—findings consistent with numerous prior studies. Our results demonstrated that posterior acoustic attenuation and PR positivity served as protective factors against high Ki-67 expression. Posterior acoustic attenuation reflects a histologic composition dominated by fibrous stroma, which is associated with lower proliferative activity (19). Such discrepancies may stem from variations in study design, sample size, or imaging assessment criteria. A substantial body of literature indicates that PR positivity denotes hormone-dependent tumor subtypes, where growth is regulated by estrogen and progesterone signaling pathways (20). These tumors typically exhibit less aggressive biological behavior with slower cell proliferation, resulting in lower Ki-67 expression (21, 22). Collectively, the clinical prediction model constructed using these factors provides a clinically applicable tool for the non-invasive preoperative assessment of breast cancer proliferative activity and invasive potential.

Traditional radiomics approaches are largely confined to extracting features from the entire tumor, failing to effectively capture intratumoral spatial heterogeneity. In this study, we successfully quantified tumor heterogeneity using habitat imaging technology. By applying the K-means clustering algorithm, we partitioned tumors into four subregions with distinct imaging phenotypes and characterized the spatial heterogeneity of the tumor microenvironment through multi-dimensional quantification of local entropy and energy values derived from each subregion.

Our results demonstrated that the habitat model achieved AUC values of 0.814 and 0.745 in the training and validation sets, respectively, confirming a significant association between tumor spatial heterogeneity features and Ki-67 expression status. And Habitat2_wavelet_HH_firstorder_Uniformity utilizes high-frequency wavelet components to capture texture uniformity variations caused by dense cellular packing; low uniformity often correlates with complex mitotic edges in high-proliferation zones. Habitat3_exponential_glszm_ZoneVariance reflects structural disorganization within tumors where necrotic and actively proliferating zones coexist, using grayscale zone variance after exponential enhancement. High variance indicates the uneven growth and microenvironmental imbalance typical of high Ki-67 tumors; Habitat2_squareroot_firstorder_Kurtosis identifies extreme outliers in intensity distribution by recognizing kurtosis after dynamic range compression. High proliferative activity often accompanies dramatic fluctuations in local metabolism or density (heavy-tailed distribution). Combining these three metrics constructs a radiomics fingerprint for Ki-67 high-expression states across three dimensions: texture consistency, structural dispersion, and intensity extremity. This finding aligns with previous studies: Li (23) found that tumor subregion features identified via MRI habitat analysis were closely correlated with breast cancer molecular subtypes, highlighting the unique advantages of habitat imaging in quantifying tumor heterogeneity. Additionally, Chen et al. explored the value of intravoxel incoherent motion (IVIM)-based habitat imaging in predicting immunohistochemical markers in breast cancer patients, further supporting the utility of this approach in capturing tumor biological characteristics (24).

Our study demonstrated that the multimodal feature fusion strategy significantly enhanced the predictive efficacy of the model. By integrating clinicopathological parameters (e.g., progesterone receptor [PR] status, axillary lymph node metastasis [LMN]) with habitat radiomics features, the Clinics_Habitat_Radiomics model achieved an area under the receiver operating characteristic curve (AUC) of 0.877 in the training set. Calibration curve analysis revealed excellent agreement between predicted probabilities and actual observations, with a Hosmer-Lemeshow test result indicating no significant deviation from perfect calibration (P> 0.05). Notably, decision curve analysis (DCA) highlighted the model’s superior clinical utility: it conferred a higher net clinical benefit than single-modality models across a threshold probability range of 20%–65%. This finding underscores the model’s potential to guide clinical decision-making more effectively than approaches relying solely on clinical or radiomic features. Our results align with those of Cai et al. (25), who reported that combining clinicopathological and radiomics features improved the predictive accuracy of neoadjuvant chemotherapy response in breast cancer—a testament to the generalizability of multimodal fusion strategies in precision oncology.

In this study, we employed a dual-feature selection strategy combining minimum redundancy maximum relevance (mRMR) and random forest (RF) algorithms to optimize feature extraction from 1,312 original variables. This approach identified a robust subset of predictors with maximal discriminative power. SHAP value analysis further revealed that habitat-derived intratumoral features contributed significantly more to model performance compared to conventional single-region intratumoral features, highlighting the value of capturing spatial heterogeneity within the tumor. This observation underscores the hypothesis that tumor microenvironmental heterogeneity may harbor critical biological insights into Ki-67 expression regulation, potentially reshaping our understanding of tumor biology and therapeutic response mechanisms.

This study has several limitations that should be acknowledged. First, the use of a homogeneous patient cohort from a single institution and standardized imaging protocols may introduce selection bias, limiting the generalizability of the model to broader clinical settings. Second, the exclusive reliance on 2D grayscale ultrasound images precluded the acquisition of 3D volumetric data, which may have introduced sampling errors and compromised the comprehensive assessment of tumor heterogeneity and biological characteristics. Third, although the sample size of 288 patients was sufficient for preliminary modeling, it was inadequate to support the application of complex algorithms such as deep learning, and there remains a risk of overfitting. A slight decline in performance was observed in the validation set (AUC 0.830), suggesting that the model may have overfit to specific features in the training data compared to the training set (AUC 0.877). This discrepancy may also indicate that the current model has not yet fully achieved generalization. Therefore, these findings underscore the need for external validation using larger, multicenter datasets to rigorously assess the model’s stability and true generalization capability.

Future research should prioritize multicenter, prospective studies incorporating 3D ultrasound imaging of breast masses and multimodal ultrasound techniques to enhance the model’s generalizability and robustness, thereby further improving the predictive capability for Ki-67 expression in breast cancer.

## Conclusion

This study pioneered the development and validation of a habitat-based ultrasound radiomics nomogram for noninvasive prediction of Ki-67 expression status in breast cancer. The tool demonstrates robust predictive accuracy, enabling dynamic monitoring of tumor proliferative activity without invasive procedures. By integrating spatial heterogeneity metrics derived from tumor microenvironment characterization, this nomogram provides a clinically actionable framework for optimizing individualized treatment regimens and guiding therapeutic decision-making in breast cancer management.

## Data Availability

The original contributions presented in the study are included in the article/**Supplementary Material**. Further inquiries can be directed to the corresponding author.
