# Supplementary material for: Ultrasound-based radiomics and habitat analysis for noninvasive assessment of Ki-67 overexpression in breast cancer
Source: Front Oncol. 2026 Apr 29;16:1803826. doi: 10.3389/fonc.2026.1803826 (PMC13167502; doi:10.3389/fonc.2026.1803826)
Supplement: Supplementary file 1 [file Table1.docx]

**Supplementary Materials**

**Supplementary Data S1.** Derivation of Clinical Prediction Model

Risk Score = 1.511430297999127 – 1.1261270354741266 × PR + 0.7507276502626985 × Lymph node metastasis – 1.20868704120921 × Orientation - 0.8946737906804668 × Posterior features

**Supplementary Data S2.** Model performance of each cohort

| Model | Model | AUC (95% CI) | ACC | TPR | TNR | PPV | NPV | BER |
| --- | --- | --- | --- | --- | --- | --- | --- | --- |
| Training cohort | Clinics | 0.729(0.657-0.8) | 0.652 | 0.604 | 0.746 | 0.827 | 0.485 | 0.325 |
|  | Habitat | 0.814(0.951-0.877) | 0.682 | 0.567 | 0.910 | 0.927 | 0.513 | 0.261 |
|  | Radiomics | 0.753(0.681-0.825) | 0.657 | 0.597 | 0.776 | 0.842 | 0.491 | 0.313 |
|  | Clinics_Habitat | 0.849(0.791-0.906) | 0.711 | 0.657 | 0.821 | 0.880 | 0.545 | 0.261 |
|  | Clinics_Radiomics | 0.815(0.754-0.877) | 0.697 | 0.619 | 0.851 | 0.892 | 0.528 | 0.265 |
|  | Clinics_Habitat_Radiomics | 0.877(0.826-0.929) | 0.746 | 0.679 | 0.881 | 0.919 | 0.578 | 0.220 |
| Validation cohort | Clinics | 0.749(0.642-0.856) | 0.632 | 0.571 | 0.792 | 0.878 | 0.413 | 0.318 |
|  | Habitat | 0.745(0.626-0.864) | 0.552 | 0.429 | 0.875 | 0.900 | 0.368 | 0.348 |
|  | Radiomics | 0.742(0.615-0.868) | 0.644 | 0.587 | 0.792 | 0.881 | 0.422 | 0.311 |
|  | Clinics_Habitat | 0.81(0.705-0.916) | 0.609 | 0.508 | 0.875 | 0.914 | 0.404 | 0.306 |
|  | Clinics_Radiomics | 0.803(0.693-0.913) | 0.632 | 0.556 | 0.833 | 0.898 | 0.417 | 0.306 |
|  | Clinics_Habitat_Radiomics | 0.83(0.792-0.93) | 0.690 | 0.603 | 0.917 | 0.950 | 0.468 | 0.240 |

**Supplementary Data S3.** The results of the DeLong test in the training cohort.

| Model | Model | P | Z |
| --- | --- | --- | --- |
| Clinics_Habitat_Radiomics | Clinics | <0.001 | -4.810 |
|  | Habitat | 0.006 | -2.734 |
|  | Radiomics | <0.001 | -3.6651 |

**Supplementary Data S4.** The results of the DeLong test in the validation cohort.

| Model | Model | P | Z |
| --- | --- | --- | --- |
| Clinics_Habitat_Radiomics | Clinics | 0.083 | -1.736 |
|  | Habitat | 0.030 | -2.173 |
|  | Radiomics | 0.063 | -1.860 |
